# Supplementary material for: Interplay of Impaired Cellular Bioenergetics and Autophagy in PMM2-CDG
Source: Genes (Basel). 2023 Aug 4;14(8):1585. doi: 10.3390/genes14081585 (PMC10454768; doi:10.3390/genes14081585)
Supplement: Supplementary file 1 [file genes-14-01585-s001.zip › Supplementary Table 3 and 4.pdf]

	Controls (n=3)			*PMM2-CDG (n=8)*							
Maximal Respiration	0.91	1.18	0.9	0.95	0.56	0.69	0.48	0.39	0.77	0.82	0.51
ATP- linked Respiration	0.97	0.99	1.13	1.32	0.64	0.64	0.48	0.44	0.9	0.98	0.69

Supplementary Table 1. Datapoints of maximal respiration and ATP linked respiration as measured by the MitoStress assay.

	Controls (n=5)					PMM2-CDG (n=8)							
glycoATP production rate (pmol/min)	0.83	0.85	1.22	0.84	1.26	0.97	0.70	0.47	0.61	0.72	0.85	0.91	0.38
mitoATP production rate (pmol/min)	1.09	1.51	0.81	1.08	0.51	0.96	0.42	1.25	0.87	0.78	1.53	1.31	1.22
total ATP production rate (pmol/min)	0.90	1.02	1.11	0.91	1.06	0.97	0.63	0.67	0.68	0.73	1.03	1.01	0.60
XF ATP rate index	1.20	1.70	0.58	1.17	0.35	0.96	0.48	2.46	1.22	0.99	1.60	1.32	3.34
% of glycolysis	0.92	0.84	1.10	0.93	1.21	1.01	1.16	0.69	0.92	1.00	0.83	0.89	0.68
% of oxidative phosphorylation	1.21	1.44	0.72	1.19	0.43	0.98	0.56	1.84	1.22	1.00	1.45	1.29	1.86

Supplementary Table 2. Datapoints of glycoATP, mitoATP and total ATP production rates, XF ATP rate Index,% of glycolysis, % of oxidative phosphorylation as measured by the MitoStress assay.
